# Toxicological Approach in Chronic Exposure to Lead on Reproductive Functions in Female Rats (*Rattus Norvegicus*)

**DOI:** 10.4103/0971-6580.68340

**Published:** 2010

**Authors:** V. Dhir, P. Dhand

**Affiliations:** Assistant Professor, Post Graduate Department of Chemistry, G.H.G Khalsa College, Gurusar Sardhar-Ludhiana-141004 (Affiliated to Punjab University, Chandigarh), India; 1 Ex Research Scholar, Department of Zoology, Punjab Agricultural University, Ludhiana - 141 001, India

**Keywords:** Proteins, acid Phosphatase, alkaline Phosphatase, alanine Aminotransferase, and Aspartate Aminotransferase

## Abstract

Lead being a toxic cumulative poison and an environmental pollutant, experiments were conducted at an oral chronic dose of (60 mg/kg/day) for 90 days on adult female rats (*Rattus Norvegicus*) and its effect on the reproductive functions in relation to the biochemical effects was studied. It was observed that the chronic dose of lead caused an elevation in the level of proteins, acid phosphatase, alkaline phosphatase, alanine aminotransferase, and aspartate aminotransferase in all the soft tissues studied indicating tissue damage. It also inhibited the level of acetylcholinesterase in all the tissues. Fertility tests by pairing treated females with males showed that lead-treated female showed irregular estrous cycle and the fertility rate dropped to 40% as female pups of lead-treated mothers showed loss in weight, high mortality rate, poor growth rate, and late vaginal opening. Histological studies of ovary showed atresia in all the stages of folliculogenesis sustaining the poor fertility observations. The present study revealed that lead caused great tissue damage and affected reproductive performance of female rats at a chronic dose.

## INTRODUCTION

Heavy metal toxicity is a serious worldwide problem, which adversely affects the growth, health, reproductive performance, and life span of all living organisms.[1] Lead is known to be toxic when present in traces and enters human body as a result of environmental pollution.[2] Occupational hazards due to lead exposure produce reversible changes in mood and personality as fatigue, irritability, depression, deficits in vascular motor functioning, memory, and verbal ability.[3] Children exposed to lead are reported to have adverse effects on central nervous system and kidneys.[4] Maternal blood lead level as an environmental factor is an apparent predictor of low birth weight and child body mass ratio[5] and low to moderate environmental exposure increases the risk for spontaneous abortion.[6] Anemia, which is frequently observed in lead poisoning, was a result of decreasing lifetime of erythrocytes and synthesis of heme.[7] In Ludhiana (Punjab, India), the analysis of water samples of Budha Nallah after the input of effluents by dying industries and pesticide manufacturing units indicates that the concentration of lead has increased manifold[8] and the mean daily intake of lead was 162.32±19.1 *μ*g/day.[4]

Lead has no known biological function and any lead absorbed by man or animals may be potentially toxic. All spheres which are affected by lead can cause 33% increase in absorption of lead, which interferes with blood-forming processes, vitamin D metabolism, and other kidney and neurological processes.[9] Lead has high affinity for various complexing groups such as imidazole, cysteine sulfhydryls, and amino group of lysine. By complexing with these moieties, lead may interfere with biochemical processors through alterations of structural integrity of enzyme or by disruption of substrate binding. The toxic effects are many, ranging from morphological tissue damage at higher concentration to lesser biochemical effects at lower concentrations.[10] Mating involving one lead toxic parent have recorded significant decrease in litter size, birth weight, and survival rate.[11,12] A variation in the time of vaginal opening and a significant disturbed estrous cycle was also observed in lead toxicity.[13] Since the absorption of lead-indicated toxicity in humans is great due to the intake through food, air, and water, it became imperative to carry out a systematic study on the effect of chronic oral dose of lead on female reproductive functions and also to record the various enzymatic changes in rats. These findings would be useful in understanding the various effects on sensitive species and also extrapolating, with care, the results for humans.

## MATERIALS AND METHODS

Disease-free albino rats 2–3 months were procured from Small Animal’s Colony, Department of Zoology, Punjab Agricultural University, Ludhiana, India. All the animals were maintained on rat feed (Ashirwad Industries, Chandigarh-India) and black gram. Water was provided *ad libitum*. Blood samples were drawn into heparinized tubes and plasma was separated after centrifugation at 3000 rpm for 5 minutes at room temperature. The plasma was diluted in the ratio of 1:10. The tissue samples were homogenized in the homogenizer in potassium phosphate buffer in the ratio of 1:10. The effect of lead on aspartate aminotransferase, alanine aminotransferase, acid phosphate, and alkaline phosphatise was estimated by the method of Wootton.[14] The cholinesterase activity was determined according to the method of Voss and Sachsse[15] and total proteins were determined by Lowry *et a*.[16] Statistical significance of biochemical parameters was obtained by students *t*-tests at 1% level (*P*<0.01) and at the 5% level (*P*<0.05).

State of the estrous cycle of each animal was determined by taking vaginal smears[17] daily between 9:30 a.m. to 10:30 a.m. In order to take vaginal smears, the vaginal was washed with physiological saline (0.9%) by injecting a drop of solution with a dropper. The vaginal smears were examined immediately under the microscope while still wet and the cellular components were judged to determine the various stages of estrous cycle with the help of following criteria: diestrus – leucocytes only; proestrus – epithelial cells with nuclei; estrus – vaginal cornification with total absence of leucocytes; and metoestrus – leucocytes with few cornified epithelial cells. For histopathological study, a piece of ovary was fixed for 24 hours in alcoholic bouins fluid. The animals were sacrificed at 30, 60, 90 days after dose administration and ovaries were removed, cleaned of adjoining tissues, and fixed in alcoholic bouins solution. The tissue was then processed for histological studies and serial paraffin sections were cut at 7 *μ*m. These sections were stained with hematoxylin and eosin and stained serial section of ovaries were examined under light microscope and morphological characteristics of normal and arteric follicles observed.

Fertility tests were conducted by treating female rats continuously for 3 months with lead (60 mg/kg/day) and housed with mature normal untreated males. The males were separated from females after formation of vaginal plug. The female were observed for entire gestation period of 28 days and the parameters of birth rate, litter size, morphological alterations, survival rate of pups, body weight from birth to 60 days, and vaginal opening in female pups for the litter were recorded. The surviving pups were then administrated lead at a rate of 60 mg/kg body weight after weighing up to 60 days of age.

## RESULTS AND DISCUSSIONS

### Biochemical parameters

Daily oral administration of lead (60 mg/kg/day) for 90 days produced a significant rise in the levels of acid phophatase in lever, kidney, and ovary and a nonsignificant increase in enzyme in plasma following daily exposure to lead. Acid phosphatase is a lysosomal enzyme and is stimulated in cases of tissue damage.[18] Increase in level of acid phosphatase in liver and kidney might be suggestive of increasing physiological phagocytosis[19] and the moderate amount of acid phosphatase activity in regressing luteal cells of the ovary indicated lysosomal activity in luteolysis.[20] The increase in acid phosphatase activity estimated biochemically would, therefore, mean a destruction of the luteal cells, which is in support of the fact that absence of acetylecholinesterase activity in ovary also causes lack of steroidogenesis. Ryan[21] had also associated a relationship of acid phosphatase being a lysosomal enzyme playing a phagocytic role in follicle cells during atresia. It has been further suggested that in follicle cells, lysosomal enzymes affect estrogen receptor by dephosphorylation, which led to atresia and also the enzyme acid phosphatase is an excellent indicator of atrophy.[22,23]

Lead (60 mg/kg/day) caused a significant increase in alkaline phosphatase level [Table 1] in plasma, liver, kidney, and ovary. It has been suggested that an increase in alkaline phosphatase level occurs due to the damage of the cells of liver, kidney, small intestine, and bone resulting in the liberation of this enzyme in the blood systems.[24] Gouda *et Al*.[25] recorded an increase in the value of alkaline phosphatase in lead toxicosis in adult goats. Alkaline phosphatase helps in ionic movement across the cell membrane and is also associated with secretory and absorption processes of the cell.[26] Wise[23] in bovine follicles also postulated AKP as an excellent indicator of atresia since AKP activity was greater in ovary. Goody *et al*.[27] reported that there was a direct or indirect evidence of the role of AKP in steroid receptor inactivation in the granulose cells. The changes in enzymes system had been correlated with the steroid biosynthesis in the granulose cells of maturing follicles of mammalian ovary.[28]

**Table 1:** Effect of lead on tissue phosphatases

Organ	Control	Treatment					
		15 days	30 days	45 days	60 days	75 days	90 days
Acid phosphatase (*n* mol phenol liberated/min/ml) (Mean±S.D.)							
Plasma	0.691±0.2001	0.622±0.092	0.656±0.061	0.670±0.273	0.699±0.158	0.715±0.099	0.729±0.043
Liver	118.93±2.95	119.41±1.92	116.43±2.48	130.72±0.97^a^^b^	125.30±2.69^a^	147.92±2.40^a^^b^	196.52±3.69^a^^b^
Kidney	9.315±0.258	9.283±2.240	15.590±3.120^a^^b^	26.326±1.77^a^^b^	26.058±2.880^a^^b^	25.550±0.938^a^^b^	29.055±1.301^a^^b^
Ovary	4.069±0.65	4.527±0.078	4.222±0.056	4.54±0.403	5.73±0.698	9.71±0.146^a^^b^	21.934±0.639^a^^b^
Alkaline phosphatase (*n* mol phenol liberated/min/ml) (Mean±S.D.)							
Plasma	13.81±0.215	12.609±0.880^a^	18.487±0.955^a^^b^	22.214±1.090^a^^b^	24.535±1.190^a^^b^	29.54±0.455^a^^b^	40.912±0.346
Liver	27.15±0.786	27.950±0.673	29.530±0.600^a^^b^	35.091±1.630^a^^b^	30.630±0.304^a^^b^	35.841±1.013^a^^b^	42.349±1.960^a^^b^
Kidney	1630.003±12.930	1846.310±24.140^a^^b^	1857.760±20.980^a^^b^	1801.551±18.490^a^^b^	1874.277±39.950^a^^b^	1964.194±21.380^a^^b^	2846.250±19.330^a^^b^
Ovary	12.193±3.050	14.280±0.495	21.550±7.690	22.261±2.480^a^^b^	26.998±2.970^a^^b^	31.460±0.500^a^^b^	45.260±9.900^a^^b^

a Statistically significant difference (*P*<0.05) when compared to values of control animals;

b Statistically significant difference (*P*<0.01) when compared to control animals; All values given are the mean of three animals except control; Control values given are the mean of four animals

Lead at the dose rate of 60 mg/kg/day for 90 days produced an overall increase in the levels of alanine aminotransferase in plasma, liver, and ovaryand a nonsignificant rise in its level in kidney [Table 2]. Alanine aminotransferase is present in liver, kidney, heart, skeletal muscles, intestines, and RBC[29] and its increased values are specific indicator of hepatocellular (liver) damage.[30] Lead also produced significant increase in aspartate aminotransferase in liver, plasma, and ovary [Table 2]. Aspartate aminotransferase SGOT occurs mainly in muscles[29] and increases in its activity related to the leakage of enzyme from muscles because of muscular activity induced by intoxication. Direct effect of lead on muscles increasing the permeability of cell membrane cannot be excluded.[31] Elevation of both alanine and aspartate aminotransferases in blood had been used also as an indicator of altered permeability of plasma membrane,[32] cellular damage,[33] and altered metabolism during insecticide toxicity.[34]

**Table 2:** Effect of lead on tissue aminotransferases

Organ	Control	Treatment					
		15 days	30 days	45 days	60 days	75 days	90 days
Alanine aminotransferase (*n* mol pyruvate formed/min/ml) (Mean±S.D.)							
Plasma	12.22±0.22	3.9300±0.187^a^^b^	12.880±0.160^a^^b^	12.966±0.050^a^^b^	13.430±0.720^a^^b^	15.420±0.260^a^^b^	19.315±0.410^a^^b^
Liver	428.310±19.64	570.146±16.07^a^^b^	658.940±5.380^a^^b^	477.900±21.17^a^	491.070±2.570^a^^b^	541.090±40.17^a^^b^	566.090±11.56^a^^b^
Kidney	52.400±10.73	55.240±0.650	59.930±4.480	60.500±13.09	58.600±8.900	65.620±6.250	68.996±9.610
Ovary	6.560±0.180	9.490±0.310^a^^b^	15.87±8.650^a^^b^	17.47±10.30^a^^b^	24.78±0.760^a^^b^	20.83±5.480^a^^b^	31.83±0.630^a^^b^
Aspartate aminotransferase (*n* mol pyruvate formed/min/ml) (Mean±S.D.)							
Plasma	2.15±0.04	5.09±0.26^a^^b^	4.30±0.79^a^^b^	5.93±0.53^a^^b^	5.92±0.77^a^^b^	5.78±0.05^a^^b^	6.99±1.02^a^^b^
Liver	263.10±21.57	275.91±12.57	294.82±9.210	299.92±12.32^a^	298.03±11.24	304.44±33.62	313.81±26.74^a^^b^
Kidney	209.60±10.11	231.94±13.85	263.41±2.050^a^^b^	292.75±11.56^a^^b^	294.07±13.82^a^^b^	319.76±12.27^a^^b^	344.35±14.41^a^^b^
Ovary	4.03±0.74	4.140±0.62	7.120±0.49^a^^b^	7.520±0.13^a^^b^	12.93±0.58^a^^b^	15.15±2.05^a^^b^	22.74±1.74^a^^b^

a Statistically significant difference (*P*<0.05) when compared to values of control animals;

b Statistically significantly difference (*P*<0.01) when compared to control animals; All values given are the mean of three animals except control; Control values given are the mean of four animals

Daily oral administration of lead produced significant decrease in AChE level in liver, kidney, and ovary and nonsignificant decrease in plasma [Table 3]. The decrease in activity of acetylcholinesterase observed in present study was similar to that recorded by Setia *et al*.[35] in calves. It was considered that decrease in AchE activity was responsible for behavioral and locomotor changes recorded in lead-intoxicated calves.[36] Acetylcholinesterase in the luteal cells of ovary hydrolyses acetylcholine in the production of acetic acid, which is used subsequently in the pathway for the production of steroidogenes for hormone production in the goat ovary.[37] Thus decrease in AChE activity in the rat ovary might be an indicator of the lack of steroidogenesis resulting in poor fertility.

Significant increase in the levels of proteins in liver, kidney, ovary and nonsignificant increase in proteins in plasma were observed following daily oral dosing of lead [Table 3]. The elevation of proteins is reported to occur in conditions when the cells are subjected to wide variety of environmental assaults including toxins, poisons, and pollutants and is mainly due to stimulation of the synthesis of acute phase protein and corresponding m-RNA,[38] which buffer them from harm.[39] Elevation of proteins might also be due to destruction of tissues, which causes release of proteins.

**Table 3:** Effect of lead on tissue acetylcholinesterase and proteins

Organ	Control	Treatment					
		15 days	30 days	45 days	60 days	75 days	90 days
Acetylcholinesterase (*n* mol acetylcholine hydrolyzed/min/ml) (Mean ± S.D.)							
Plasma	0.1075±0.09	0.045±0.029	0.060±0.030	0.070±0.024	0.090±0.080	0.076±0.067	0.056±0.070
Liver	3.70±0.56	0.500±0.050^a^^b^	0.820±0.088^a^^b^	0.647±0.068^a^^b^	0.552±0.070^a^^b^	0.909±0.066^a^^b^	0.713±0.050^a^^b^
Kidney	0.560±0.05	0.417±0.064^a^	0.263±0.010^a^^b^	0.464±0.073^a^^b^	0.246±0.095^a^^b^	0.310±0.048^a^^b^	0.156±0.034^a^^b^
Ovary	0.459±0.07	0.257±0.057^a^^b^	0.157±0.040^a^^b^	0.367±0.047	0.388±0.047	0.275±0.017^a^^b^	0.149±0.090^a^^b^
Proteins (g/100 ml) (Mean ± S.D.)							
Plasma	0.77±0.096	0.076±0.005	0.079±0.004	0.082±0.008	0.086±0.002	0.081±0.077	0.092±0.033
Liver	0.385±0.0176	0.381±0.010	0.388±0.023	0.450±0.020^a^^b^	0.476±0.052^a^^b^	0.437±0.012^a^^b^	0.441±0.002^a^^b^
Kidney	0.298±0.016	0.284±0.004	0.286±0.032	0.403±0.057^a^	0.464±0.016^a^^b^	0.470±0.025^a^^b^	0.477±0.011^a^^b^
Ovary	0.0715±0.038	0.0710±0.0049	0.094±0.0125	0.098±0.004	0.154±0.036^a^^b^	0.161±0.004^a^^b^	0.170±0.023^a^^b^

a Statistically significant difference (*P*<0.05) when compared to values of control animals;

b Statistically significantly difference (*P*<0.01) when compared to control animals; All values given are the mean of three animals except control; Control values given are the mean of four animals

### Fertility test

**Figure 1:**
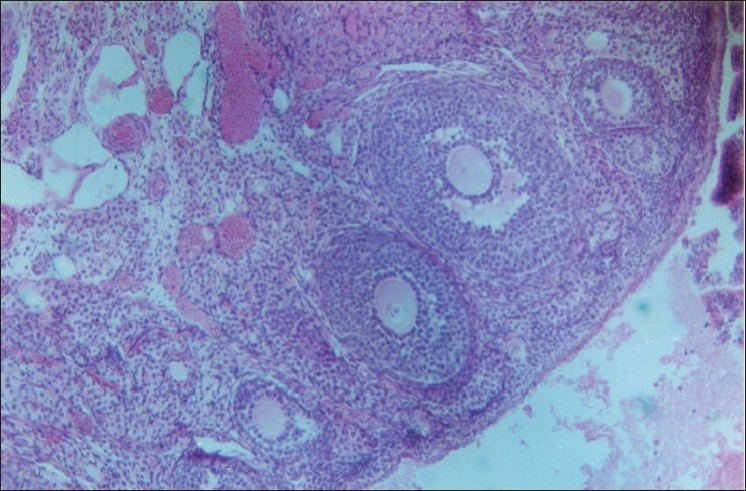
Various stages of follicles undergoing atrsia (HE stain) 100×

**Figure 2:**
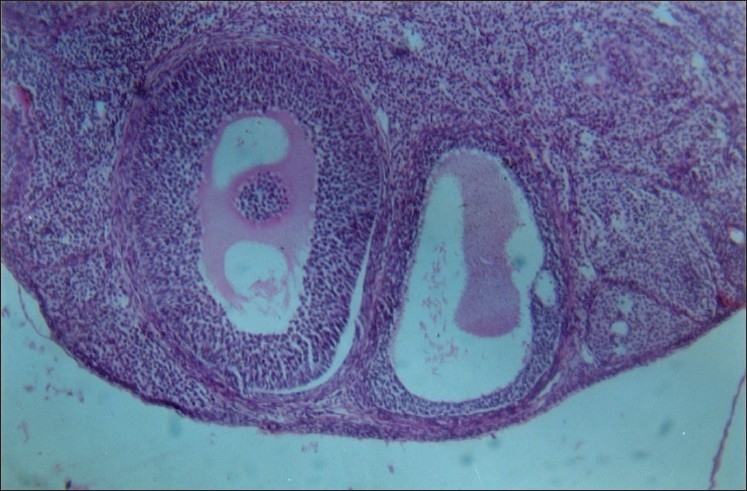
Incipient antral stage follicle undergoing atresia (HE stain) 100×

**Figure 3:**
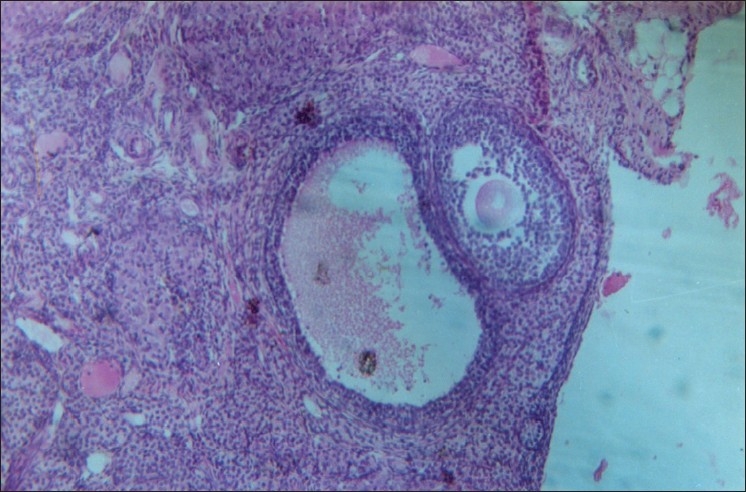
Antrum formed Graafian follicle undergoing atresia with complete detachment of granulose from theca shows advanced stage in atresia (HE stain) 100×

**Figure 4:**
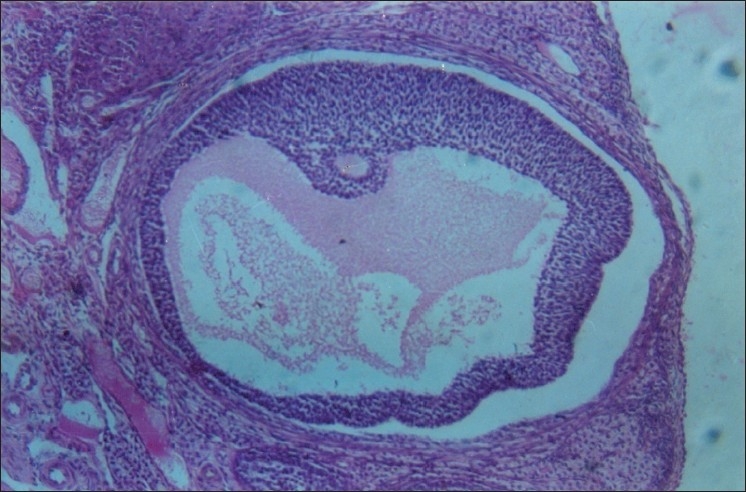
Antrum formed Graafian follicle undergoing atresia with complete detachment (arrowheads) of granulose from theca shows advanced stage in atresia (HE stain) 100×

Five sets of experiments that were set up for the testing effect of lead on fertility of rats indicated that lead at a dose of 60 mg/kg caused 40% reduction in the fertility rate [Table 4] as compared to control group of rats, which showed 100% results. The decrease in fertility has been related to the decrease in AChE concentration, which is considered important in the process of steroidogenesis, and increase in the level of other enzymes, which might be damaging to the tissue leading to atresia [Figures 1–4]. Most reproductive functions are controlled by sex steroids, the possibility that changes in the synthesis/breakdown of these hormones may alter reproductive capacity in man and other animals exposed to lead cannot be excluded. Odland *et al*.[5] reported that exposure to organic lead affects the metabolism of steroid hormones in human and mice. Implantation of the blastocyst in the uterine endometrium requires a delicate balance between progesterone and estrogens.[40] Chronic dosage of lead probably imbalances this delicate interplay of hormones and disallows implantation in rat.

In addition to the observations made above, the treated females showed irregularity in estrous cycle. Der *et al*.[41] and Ronis *et al*.[13] have also reported irregularity in estrous cycle of female albino rats. Female pups of treated mother also showed late vaginal opening, poor fur growth, significantly lower body weight [Table 5], and decrease fetal survival ratio [Table 6]. Ronis *et al*.[11,12] also reported that pups of treated mothers have high mortality rate and lead also effects growth rate. Maternal–fetal transfer of nutrients is an established phenomenon and death of young ones of lead-poisoned mothers could represent the placental transfer of lead. Such an observation has been reported by Singh *et al*.[42] Lead-fed rats showed significant decrease in body weight [Table 7]. Lower body weight of rats fed lead in diet as compared to control rats has been well advocated by Ryden and Walsh[21] and Schwark *et al*.[43] Furthermore environmental toxicants, teratogenic compounds can have drastic affects on the survival rate of embryos when ingested at crucial early stages of gestation.[44]

The above study concluded that lead has interaction with the vital body functions and reproductive parameters in rats. The dosage administered caused significant biochemical alterations and reduction in the weight of pups as well as the treated mothers. Lead caused high mortality rate in pups and also slows down their growth rate.

**Table 4:** Fertility assessment of lead-treated (60 mg/kg) female rats

Number of days of treatment	Treated (female)	Untreated (male)	parturition	Size of litter (number)	Gestation period (days)	Died at birth time (number)
90	2	1	½	11	20–22	2
90	2	1	0/2	*	*	*
90	2	1	½	8	21–24	3
90	2	1	½	9	21–23	4
90	2	1	½	7	22–24	2
0	None (control)	10 females, 3–4 males	10/10	6–10	25–27	0

* Female rats did not show any signs of pregnancy till the end of the experiment

**Table 5:** Effect of lead on body weights of pups of treated mothers and dose after lactation

Body weight at birth (mean±S.D.)		15 days	30 days	45 days	60 days
Control	7.06±0.24	18.40±1.94	40.86±3.42	59.86±2.43	81.92±4.61
Treated					
A	5.250±0.900^a,b^	11.2901.25^a,b^	21.290±2.46	35.21±0.21^a,b^	—
B	5.306±0.370^a,b^	9.82602.27^a,b^	16.440±4.29	33.80±0.39^a,b^	—
C	5.570±0.233^a,b^	10.9121.03^a,b^	20.990±1.56	31.82±0.00^a,b^	30.21±0.00^a,b^ (died on day 63)
D	5.490±0.150^a,b^	10.5601.97^a,b^	20.765±1.14	31.48±0.00^a,b^	—

**Table 6:** Survival rate of pups

Days of treatment (female)	Number of pups born	Survival at birth time	Survival after 15 days	Survival after 30 days	Survival after 30 days	Survival after 45 days	Survival after 60 days
Control (no treatment)	6–10	6–10	6–10	6–10	6–10	6–10	6–10
60	11	9	8	6	6	3	Died
60	Nil	Nil	Nil	Nil	Nil	Nil	Nil
60	8	5	5	4	4	2	Died
60	9	5	5	3	3	1	1
60	7	5	3	2	2	1	Died

**Table 7:** Effect of lead on body weight of treated female

Treatment dose (mg/kg)	0 day	15 days	30 days	45 days	60 days	75 days	90 days
0	122±4.52	132±3.33	145±2.26	151±2.61	1650±4.21	170±2.17	173±2.36
60	120±2.95	1224.32^a^^b^	125±2.26^a^^b^	130±1.56^a^^b^	127±4.56^a^^b^	125±4.03^a^^b^	126±3.92^a^^b^

a Statistically significant difference (*P*0.05) when compared to values of control animals;

b Statistically significant difference (*P*<0.01) when compared to control animals
